# Novel Implantable Cardioverter-defibrillator Lead Placement in a Patient with a Prosthetic Tricuspid Valve

**DOI:** 10.19102/icrm.2017.081103

**Published:** 2017-11-15

**Authors:** Pratik Patel, Kousik Krishnan, Sandeep Saha, Richard G. Trohman

**Affiliations:** ^1^Division of Cardiac Electrophysiology, Rush University Medical Center, Chicago, IL, USA

**Keywords:** Implantable cardioverter-defibrillator, prosthetic tricuspid valve

## Abstract

As the placement of transvenous leads across a prosthetic tricuspid valve is preferentially avoided, one must consider alternative solutions to provide necessary pacing and/or defibrillator therapy. Here, we present a case of novel placement of an implantable cardioverter-defibrillator (ICD) lead in the right atrium, in order to provide safe ICD therapy in a patient with a prosthetic tricuspid valve.

## Introduction

Implantable cardioverter-defibrillators (ICDs) are the standard of care for the prevention of sudden cardiac death in at-risk individuals. Transvenous ICD systems traditionally utilize a defibrillation lead placed in the right ventricle (RV) to produce a reliable and effective defibrillation shocking vector.^1^ Unfortunately, the placement of a lead across the tricuspid valve can be an impediment to optimal valvular and RV function. While the actual incidence and consequence of clinically significant lead-induced tricuspid valve impairment remains debatable, the occasional occurrence of severe tricuspid regurgitation cannot be questioned. In certain clinical scenarios, this may require lead removal and/or tricuspid valve surgery.^2^ In such cases, the placement of another lead across a repaired or prosthetic valve is not recommended, so alternative methods for providing ICD or pacing therapy must be pursued.^3^ In considering non-thoracotomy approaches, the emerging technologies of leadless pacemakers and subcutaneous devices may ultimately provide the optimal tools, as described by Montgomery et al.^4^ However, in this current case report, we present the placement of a dual-chamber ICD in a pacemaker-dependent patient utilizing a single-coil right defibrillation lead, an existing RV epicardial bipolar pace/sense lead, a subcutaneous coil, and a left pectoral generator. To the best of our knowledge, this approach has not been previously reported in the literature.

## Case presentation

A 67-year-old female with a past medical history of rheumatic mitral stenosis, non-ischemic dilated cardiomyopathy with chronic left ventricular systolic dysfunction, chronic renal insufficiency, and breast cancer (in remission) presented reporting weeks of progressive symptoms and with signs of right-sided heart failure. She had undergone both implantation of a single-chamber ICD in 2008 and bioprosthetic mitral valve replacement in 2012.

At the time of her presentation in 2012, the patient complained of increasing fatigue, lower extremity swelling, and abdominal distension. She had had multiple previous hospital admissions for symptomatic heart failure, and had been treated with diuresis and therapeutic paracenteses. A transthoracic echocardiogram revealed severe tricuspid regurgitation, with evidence of impingement from the RV defibrillation lead. A cardiovascular surgeon was consulted, and the patient was taken for tricuspid valve replacement. During surgery, the defibrillator lead was found to be compressing the septal leaflet, and its removal was deemed required for bioprosthetic valve replacement. The lead was cut immediately distal to the SVC coil and left in position in the right atrium. A bipolar epicardial RV pacing lead was subsequently implanted and placed in a pocket in her left upper abdominal quadrant. Epicardial patch(es) placement unfortunately was not performed. Postoperatively, the patient was noted to have developed complete atrioventricular block and atrial flutter. Her ventricular rhythm was maintained via temporary epicardial pacing wires placed at the time of her surgery. The electrophysiology service was consulted with regards to permanent pacing and defibrillation options.

**Figure 1:**
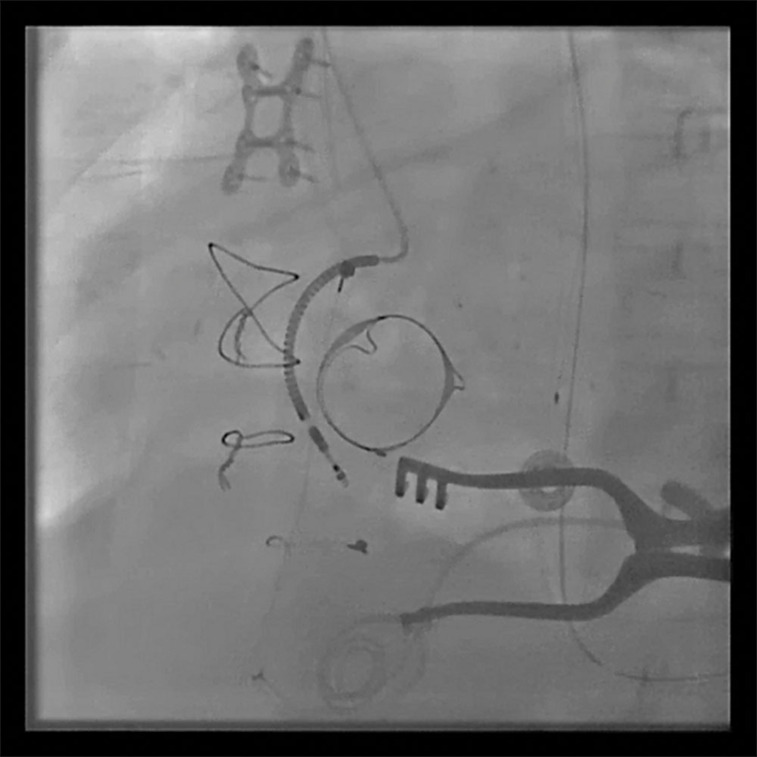
Left anterior oblique projection of the right atrial ICD lead.

**Figure 2:**
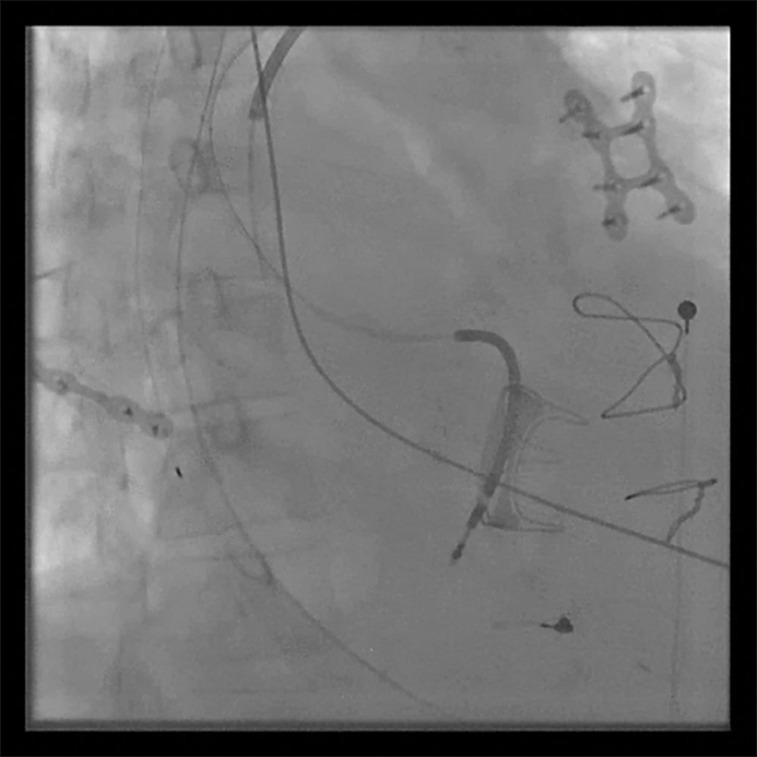
Right anterior oblique projection of the right atrial ICD lead.

**Figure 3:**
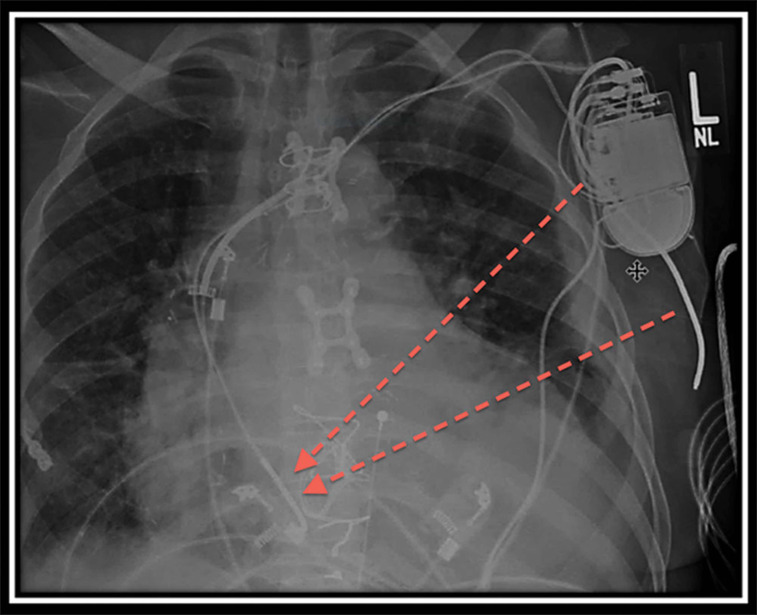
Anteroposterior chest X-ray depicting shock vector from the can/subcutaneous coil to the right atrial coil.

The patient was ultimately brought to the electrophysiology laboratory for dual-chamber ICD placement. The pre-existing left infraclavicular pocket was accessed. The RV ICD, which had been cut intraoperatively, was detached and the old ICD generator was explanted. Brief attempts to remove this lead using manual traction proved unsuccessful, so it was instead capped and left in situ. A new single-coil ICD lead (Durata™ 7122Q Defibrillation Lead; Abbott Laboratories, Chicago, IL, USA) was then placed in the right atrium as close to the tricuspid valve, and thus the RV, as possible **(Figures 1 and 2).** This lead was placed to serve as an atrial pace/sense lead and the “distal RV” ICD coil. A subcutaneous coil (Medtronic 6996SQ; Medtronic Inc., Minneapolis, MN, USA) was implanted via the left infraclavicular pocket and tunneled posterolaterally to provide a vector towards the left ventricle. The leads were attached to a dual-chamber ICD (2357-40C Fortify Assura™ DR; Abbott Laboratories, Chicago, IL, USA). Ventricular defibrillation safety margin testing was performed. The shocking configuration was set can/subcutaneous coil (cathode) to right atrium coil (anode) (**Figure 3**). Ventricular fibrillation (VF) was induced twice. There was appropriate sensing, and the patient was successfully defibrillated each time with 25 J.

## Discussion

Conventional transvenous ICD placement utilizes a right ventricular lead across the tricuspid valve. In our patient, however, this was considered to be a suboptimal option. The placement of epicardial patches at the time of valve surgery would have been ideal. We considered the increased morbidity and mortality associated with another thoracotomy very undesireable.^5^ The subcutaneous ICD provides an excellent defibrillation alterative, but the lack of pacing capability necessitated that an alternative solution be considered for use in our patient. Lead less pacemakers may prove to avoid similar issues with tricuspid valve function, but do not provide defibrillation therapy. There are few reports in the literature of non-thoracotomy approaches to lead placement that avoid crossing the tricuspid valve.

There are case reports in the literature of utilizing the coronary sinus (CS) venous system for successful placement of a defibrillation lead. Leng et al. first described the placement of a defibrillation coil in the CS in patient with a persistent left SVC and a prosthetic tricuspid valve.^6^ A middle cardiac vein (MCV) pace-sense lead and subcutaneous array were also utilized in this past case. Cohen et al. described the placement of a CS defibrillation lead with placement of the can in the abdomen.^7^ An epicardial ventricular pace-sense lead was used in their case for ventricular tachycardia/VF detection. Lopez reported a case series of six patients in whom defibrillator coil leads were successfully placed into the MCVs, and pace-sense leads either in lateral CS branches or in the atrialized RV, as was done in one patient in the series with Ebstein’s anomaly. Using the MCV-coil-to-SVC-coil/can configuration provided an adequate safety margin in five of the six patients during defibrillation threshold testing. An additional azygous coil was placed in the remaining patient. During a one- to five-year follow-up period, no instances of late complications (including lead migration) were reported.^8^ While these cases demonstrate the feasibility of defibrillation coil placement in the cardiac venous system, the risks of thrombosis, fibrosis, and/or lead migration are still somewhat unknown, due to a paucity of long-term experience. Additionally, a risk of traumatic rupture from defibrillation in the CS was demonstrated during early experiences with accessory pathway ablation using direct current shocks.^9^

There are even fewer reports of endocardial defibrillator lead placements that either do not transverse the tricuspid valve, or do not utilize the cardiac venous system. Schreiber et al. and Grimard et al. described an alternative approach in which defibrillation coils were placed free-floating in the inferior vena cava.^10,11^ Akin to our approach, Biffi et al. described the placement of a single-coil active fixation lead in the lower septal right atrium.^12^ A bipolar CS lead was used for tachycardia detection. An advantage in their case and ours, as compared with the other described cases, is a reduced risk of migration with active fixation of the defibrillator coil lead. One of the pitfalls of this approach, however, is that the right atrial lead position may be subject to change. Placement in the lateral right atrium was contemplated, but we felt that the distance to the RV was suboptimal (and could be exacerbated if the lead pulled back). We also believed that the weight of a looped ICD coil would increase the risk of lead dislodgement.

In our patient, the presence of a freshly placed tricuspid prosthesis dissuaded us from trying to access the CS. We felt that trauma to the new valve could result in the need for a repeat thoracotomy, and that the risks of this approach outweighed the potential benefits. The addition of a subcutaneous coil provided a dual-current pathway shock to ensure adequate defibrillation in this patient with severe LV dysfunction. Using an azygous vein coil, rather than a subcutaneous coil, was also considered as an option. However, this would have led to a third lead being present in the left subclavian vein system (with the cut old lead still in place). We wanted to avoid placing another transvenous lead in case lead extraction was required in the future.

Our case demonstrates a novel approach to atrial lead placement, dual-chamber pacing, and ICD therapy. Placement of a single coil in the low inferior right atrium provided an adequate shocking vector to the can and posteriorly directed subcutaneous coil. The procedure is technically straightforward, and provides an option that avoids the risk of new tricuspid valve injury.

## Conclusions

In cases where endocardial transtricuspid RV lead placement should be avoided, the placement of a defibrillation lead in the right atrium may be a viable solution when an epicardial or cardiac venous lead can provide ventricular pace/sense function. The addition of a subcutaneous axillary coil enhances the shocking vector to ensure safe defibrillation.
